# The Effects of Different Soil Component Couplings on the Methylation and Bioavailability of Mercury in Soil

**DOI:** 10.3390/toxics11110942

**Published:** 2023-11-20

**Authors:** Aming Qin, Shu Ran, Tianrong He, Deliang Yin, Yiyuan Xu

**Affiliations:** 1 Key Laboratory of Karst Georesources and Environment, Guizhou University, Ministry of Education, Guiyang 550025, China; amingqin1228@163.com (A.Q.); treshisa@163.com (S.R.); dlyin@gzu.edu.cn (D.Y.); 2 College of Resources and Environmental Engineering, Guizhou University, Guiyang 550025, China; 3 State Key Laboratory of Environmental Geochemistry, Institute of Geochemistry, Chinese Academy of Sciences, Guiyang 550081, China; xuyiyuan@mail.gyig.ac.cn

**Keywords:** soil constituents, mercury, methylmercury, bioavailability, soil texture

## Abstract

Soil composition can influence the chemical forms and bioavailability of soil mercury (Hg). However, previous studies have predominantly focused on the influence of individual components on the biogeochemical behavior of soil Hg, while the influence of various component interactions among several individual factors remain unclear. In this study, artificial soil was prepared by precisely regulating its components, and a controlled potted experiment was conducted to investigate the influence of various organic and inorganic constituents, as well as different soil textures resulting from their coupling, on soil Hg methylation and its bioavailability. Our findings show that inorganic components in the soils primarily exhibit adsorption and fixation effects on Hg, thereby reducing the accumulation of total mercury (THg) and methylmercury (MeHg) in plants. It is noteworthy that iron sulfide simultaneously resulted in an increase in soil MeHg concentration (277%). Concentrations of THg and MeHg in soil with peat were lower in rice but greater in spinach. A correlation analysis indicated that the size of soil particles was a crucial factor affecting the accumulation of Hg in plants. Consequently, even though fulvic acid activated soil Hg, it significantly increased the proportion of soil particles smaller than 100.8 μm, thus inhibiting the accumulation of Hg in plants, particularly reducing the concentration of THg (93%) and MeHg (85%) in water spinach. These results demonstrate that the interaction of organic and inorganic components can influence the biogeochemical behavior of soil Hg not only through their chemical properties, but also by altering the soil texture.

## 1. Introduction

Mercury (Hg) is an environmental pollutant that has gained global attention due to its high mobility and biotoxicity [1]. Owing to the anaerobic metabolism and rich organic matter in some aquatic environments such as wetlands and paddy fields, Hg tends to form more toxic methylmercury (MeHg). This compound easily accumulates and transfers through the food chain, posing threats to both ecological safety and human health [2,3,4,5,6,7]. Under similar pollution conditions, concentrations of MeHg are generally greater in paddy soils than in well-drained soils [8]. Various physical and chemical factors, such as redox potential, pH, soil components, Hg occurrence, and soil particle size, affect the methylation and bioavailability of soil Hg [5,9,10,11,12,13,14].

The chemical composition of soil is highly complex, encompassing various organic matters and minerals such as silicates, oxides, and sulfides. Different soil components have diverse effects on Hg. Iron (Fe) and manganese (Mn) oxides, along with natural clay minerals, generally possess large specific surface areas and porous structures with high cation exchange capacities, influencing the adsorption/desorption of metals [15,16,17]. Moreover, the Si-O-Al, C-O bonds and active groups on the surface of clay minerals, such as hydroxyl and amino groups, exhibit strong capacities for adsorbing and complexing metals and nonmetals [16,17]. Iron sulfides (FeS, Fe_2_SO_4_, etc.) can reduce the bioavailability of Hg by forming charged Hg(II)-polysulfides, thus inhibiting Hg methylation [18]. Organic matter plays a crucial role in the biogeochemical cycle of Hg in the soil. It serves as a carbon source for Hg-methylating bacteria and can adsorb or complex with bioavailable Hg, thereby regulating Hg’s bioavailability and methylation [19,20]. Organic matter includes humus, comprising fulvic acid (FA), humic acid (HA), and humin (HM). FA is soluble in both alkaline and acidic solutions, as well as in water, whereas HA is soluble in alkaline solutions but insoluble in water and acidic solutions. Different humic substances have varying effects on Hg; for instance, FA promotes the release and bioaccumulation of Hg in the soil [21,22], while HA has the opposite effect [23,24,25]. Some studies suggest that although HA inhibits soil Hg activity, it enhances Hg methylation through microorganism utilization, resulting in limited Hg reduction in rice. On the other hand, FA activates soil Hg but does not increase Hg content in rice [26].

Organic and inorganic colloids in the soil interact through various forces, forming organic-inorganic complexes with diverse forms and densities. Humus, metallic oxides, and phyllosilicates constitute basic soil components, mutually influencing the physicochemical properties of the soil, such as texture and pH, which in turn affect metal transport and transformation [27,28]. On the one hand, humus can alter the physicochemical properties of oxides and phyllosilicate mineral surfaces, impacting their distribution, migration, and sedimentation, as well as processes like adsorption, dissolution, and crystallization. This regulation extends to nutrient element concentrations, forms, chemical reactions, and the bioavailability of inorganic and organic contaminants in the soil. On the other hand, oxides and phyllosilicate minerals can promote the chemical decomposition and condensation of humus through processes like adsorption or catalytic actions on their surfaces. These interactions profoundly affect the migration, transformation, and environmental effects of humus within the soil [29,30]. Studies have shown that the formation of soil organic and inorganic complex can promote the formation of soil aggregates, make soil become soft without agglomeration, and change the physical and chemical properties of soil. In addition, the physical migration of organic and inorganic complex in soil causes the redistribution of soil materials, affecting the adsorption of metals on soil particles of different particle sizes [31,32,33]. However, previous studies on the effects of soil components on mercury biogeochemical behavior were mainly conducted from the perspective of individual components, with little attention paid to the interactions among several individuals factors of various components [34,35,36,37,38,39,40,41,42,43,44]. Moreover, previous related research on Hg primarily involved adding specific components to existing soil, making it challenging to isolate the effects of the same components already present in the soil [26,41,45,46,47]. This has hindered the understanding of the role of soil composition in mercury biogeochemical behavior.

In this study, pot experiments were conducted using rice and water spinach to investigate the influence of organic and inorganic constituents, as well as different soil textures resulting from their interactions among several individual factors, on the methylation and bioavailability of Hg. To eliminate interference from pre-existing soil components, various artificial soils were created by precisely controlling key components such as Fe/Mn oxides, kaolinite, calcium carbonate, organic matter (peat, fulvic acid, humic acid), and iron sulfide. This provides a new approach to studying the impact of soil composition on mercury biogeochemical cycling, and provides a good guide for the healthy development of ecological agriculture in mercury polluted areas.

## 2. Materials and Methods

### 2.1. Artificial Soil Preparation and Pot Experiment

Artificial soil was prepared by precisely adding or eliminating certain key components. Quartz sand, calcium carbonate, kaolin, iron oxide, manganese oxide, peat, peat-derived fulvic acid (PFA), peat-derived humic acid (PHA), and commercially purchased humic acid (MHA) were used to prepare the artificial soil for potting experiments. The simple variable method (briefly, it is to keep other soil components unchanged, and remove or add one of the above soil components) was employed to control the differences in the components of the artificial soil [48]. The soil composition of each treatment is shown in Table 1. The pot experiment consisted of one control group and eight treatment groups. Three replicates were conducted for each treatment group and control group.

Quartz sand, calcium carbonate, kaolin, iron oxide, and manganese oxide were purchased from Kermel Chemical Reagent Co., LTD, Tianjin, China, analytical pure. The peat was grinding, pass through 60-mesh sieve, the extraction and purification of peat-derived fulvic acid and humic acid were isolated using a common method developed by the International Humic Substances Society [49], and the detailed description is provided in the Supplementary Material. After purification, PFA and PHA solutions were concentrated by rotary evaporation at 40 °C. These were then freeze-dried to obtain PFA and PHA solids, which were crushed using a high-speed pulverizer and stored at a low temperature (AQ-180E, Naiou, Zhejiang, China). Humic acid (MHA) (powder, analytically pure, 97%) was purchased from Guangfu Fine Chemical Research Institute, Tianjin, China.

A predetermined amount (8 kg) of prepared soil was placed into a glass tank (45 cm × 30 cm × 28 cm) and mixed with a HgCl_2_ solution (2.0 mg/mL) to achieve a Hg concentration of 2.0 mg/kg. The soil was then aged for three weeks at room temperature before use. After aging, water spinach (Ipomoea aquatica Forssk) and rice seedlings (Yixiangyou 2115) were selected for cultivation in the same glass tank. Throughout the growing period, the plants were irrigated with tap water, and the culture period was approximately 60 days for water spinach, and 120 days for rice. The experiment was conducted in an indoor greenhouse on the roof of the College of Resources and Environmental Engineering, Guizhou University, where the temperature was controlled within a range of 17–25 °C.

### 2.2. Sample Collection

On the 60th day of rice and water spinach planting, 30 mL of soil overlying water was collected in a borosilicate glass bottle for THg determination. The soil samples were collected from 2–3 cm below the soil surface and placed into a 50 mL centrifuge tube, then stored at −20 °C. After freeze-drying, part is used to determine the soil particle size distribution, the rest was grinding, and passing through a 100-mesh sieve, these samples were used to determine soil Hg form and other physicochemical parameters. After soil sample collection was complete, the above-ground parts of the water spinach were collected, washed three times with tap water, and then with deionized water. The samples were then dried at a low temperature (40 °C), ground, sealed, and stored for later analysis. On the 120th day of rice planting, unhusked rice was collected, washed three times with tap water, followed by deionization. After drying at low temperature, the rice was shelled, ground, passed through a 100-mesh sieve, sealed, and stored for subsequent determination.

### 2.3. Chemical Analysis

The THg concentrations in the rice grain and water spinach were determined with a DMA-80 analyzer (Milestone, Milan, Italy). The THg concentrations in the overlying water samples were determined by cold vapor atomic fluorescence spectrometry (CVAFS) (Model III, Brooksrand^®^, Washington, DC, USA) using a method described in detail by [50,51]. Briefly, For the THg concentrations in the water samples, water samples were oxidized with 0.5% BrCl. After oxidation, NH_2_OH·HCl was added to destroy the free halogens before adding stannous chloride (SnCl_2_) to convert divalent Hg to volatile Hg0, trapped by gold sands and analyzed by CVAFS.

The MeHg concentrations in the rice grain, water spinach and soil were determined using methods that have been previously described by [52,53,54]. Briefly, the MeHg in soil was analyzed via CuSO_4_-HNO_3_-CH_2_Cl_2_ solvent extraction, water back-extraction by heating, and ethylation-GC-CVAFS techniques. The MeHg in rice grain and water spinach were analyzed via KOH digestion and the ethylation-GC-CVAFS technique. The detailed steps are provided in the Supplementary Material.

The bioavailable Hg contents in the soils were determined with the method of [55], which involved sequential selective extractions of Hg from geological solids. soluble and exchangeable Hg was extracted using 1M Mg(NO_3_)_2_ (adjusted to pH 7 with HNO_3_), specifically sorbed Hg was extracted using 1M CHCOONa (adjusted to pH 5 with CHCOOH). The sum of the concentrations of Hg from these two fractions was defined as the bioavailable Hg concentration [38].

Soil dissolved organic carbon (DOC) concentration was measured by a TOC analyzer (Vario TOC, Elementar, Frankfurt, Germany) at soil:water = 1:20. The pH was measured by Portable pH meter (SX731, Sanxin, Shanghai, China). Soil particles ware dispersed using water and ultrasonic waves, and their size distributions of the soil were measured by a laser-scattering particle analyzer (Bettersize3000Plus, Bettersize Instruments Ltd., Liaoning, China). The agitator speed was 800 r/min and the shading range was 5–15%.

### 2.4. Quality Assurance and Quality Control

Quality assurance and quality control for the analytical process were carried out using duplicates and standard reference materials (TORT-3, ERM-CC580, and GSB-11). The THg and MeHg recoveries for the standard reference material were 80–120%. The relative standard deviations (RSDs) on precision tests for all the duplicate samples varied were <15%.

Statistical analysis was carried out with SPSS 26 and Origin 2022. We first used the Kolmogorov–Smirnov test to find whether the overall data obeyed a certain normal distribution. Thereafter, the Kruskal–Wallis test or One-way analysis of variance (ANOVA) was used to test the significant differences between data from different processing groups.

## 3. Results

### 3.1. Physicochemical Properties of the Soils

**Figure 1:**
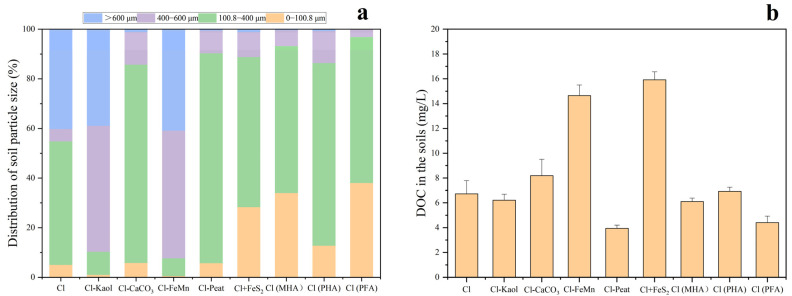
Distribution of soil particle size in each treated group (**a**), DOC in the soils (**b**). Containing all the components (control group, Cl), removal of kaolinite (Cl-Kaol), calcium carbonate (Cl-CaCO_3_), Fe/Mn oxides (Cl-FeMn) and peat (Cl-Peat); addition of ferrous disulfide (FeS_2_) (Cl+FeS_2_); replacing peat in the soil with market-derived humic acid (Cl (MHA)), peat-derived humic acid (Cl (PHA)) and peat-derived fulvic acid (Cl (PFA)).

The relative proportion of each particle size in the soils is illustrated in Figure 1a. The relative proportion of each particle size in the soil can indicate the soil texture. From the figure, it is evident that the addition or deficiency of various inorganic and organic components in the artificial soil can alter the soil texture. In comparison with the control, the soil lacking kaolinite or iron and manganese oxides both had higher the proportion of granules with a diameter of 400–600 µm. This suggests that the presence of kaolinite or iron and manganese oxides reduces the soil particle size. The treatments with addition of FeS_2_, fulvic acid and humic acid had notably higher the proportion of granules with a diameter of 0–100.8 µm. This indicates that the inclusion of FeS_2_, fulvic acid, or humic acid can refine the coarse particles in the artificial soil into finer particles. The absence of peat in the artificial soil significantly reduced the proportion of particles with sizes larger than 600 µm, suggesting that peat addition could increase soil particle size and improve soil structure.

Soil pH is slight variations in observed among each treated group, ranging from 7.34 to 7.65. Following the addition of organic matter and FeS_2_, the overall pH slightly decreased, although not significantly. DOC in the soils is presented in Figure 1b. Among all treatment groups, the soil with peat deficiency had the lowest DOC content, while the soil with iron manganese oxide deficiency and iron sulfide addition had higher DOC content.

### 3.2. The Distributions of Hg and MeHg in the Soils

**Figure 2:**
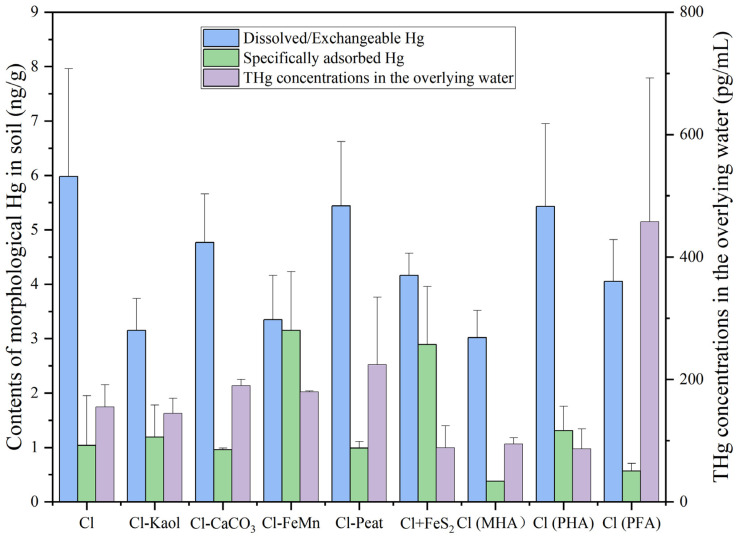
Concentrations of dissolved/exchangeable Hg and specifically adsorbed Hg in the soils (ng/g), THg concentrations in the overlying water (ng/L). Containing all the components (control group, Cl), removal of kaolinite (Cl-Kaol), calcium carbonate (Cl-CaCO_3_), Fe/Mn oxides (Cl-FeMn) and peat (Cl-Peat); addition of ferrous disulfide (FeS_2_) (Cl+FeS_2_); replacing peat in the soil with market-derived humic acid (Cl (MHA)), peat-derived humic acid (Cl (PHA)) and peat-derived fulvic acid (Cl (PFA)).

Figure 2 displays the concentrations of dissolved/exchangeable Hg and specifically adsorbed Hg in the soils, as well as THg concentrations in the overlying water of the soils. Since the overlying water and soil samples were collected during the middle stage of planting, the contents of THg in the overlying water can partially indicate the transferability of soil Hg at the early planting stage, while dissolved/exchangeable, and specifically adsorbed Hg indicate the potential for Hg release.

The concentration of THg in the overlying water in the control was 155 ng/L. In comparison with the control, the contents of THg in the overlying water of the treated group with a deficiency of kaolinite, calcium carbonate, and iron-manganese oxides showed minimal difference. Adding FeS_2_ to the control and replacing peat with humic acid both reduced the concentration of THg in the overlying water, although the difference was not significant. Conversely, the treatment with fulvic acid had significantly higher THg concentration in the overlying water (*p* < 0.05).

In the control, the contents of dissolved/exchangeable Hg and specifically adsorbed Hg were 6 ng/g and 1 ng/g, respectively. In comparison with the control, the content of dissolved/exchangeable Hg in the treated group with a deficiency of kaolinite significantly reduced by 47% (*p* < 0.05), while specifically adsorbed Hg displayed no significant difference. When the soil was deficient in iron and manganese oxides, the content of specifically adsorbed Hg significantly increased by 201%, while the content of dissolved/exchangeable Hg reduced significantly (44%). In comparison with the control, no significant difference was observed in the treated groups lacking calcium carbonate and peat regarding the contents of Hg in these two forms, similar to the scenario when replacing peat with peat-derived fulvic acid and humic acid. However, replacing peat in the soil with commercially purchased humic acid significantly reduced the concentration of dissolved/exchangeable Hg (50%) (*p* < 0.05).

**Figure 3:**
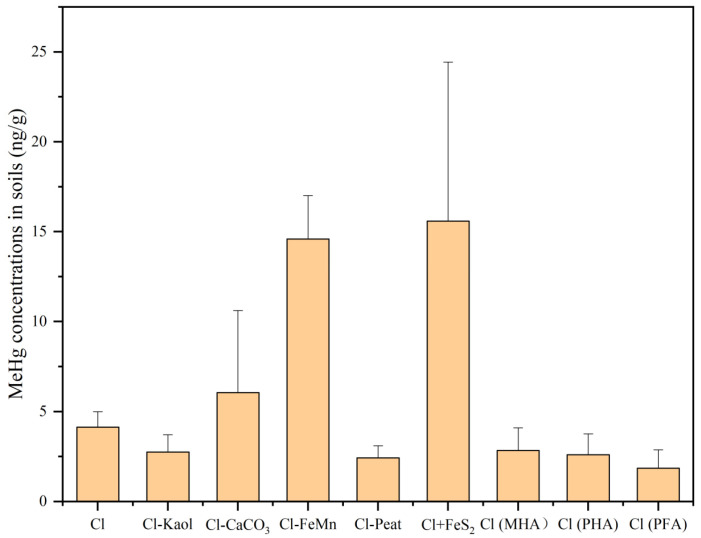
Concentrations of MeHg in the soils (ng/g). Containing all the components (control group, Cl), removal of kaolinite (Cl-Kaol), calcium carbonate (Cl-CaCO_3_), Fe/Mn oxides (Cl-FeMn) and peat (Cl-Peat); addition of ferrous disulfide (FeS_2_) (Cl+FeS_2_); replacing peat in the soil with market-derived humic acid (Cl (MHA)), peat-derived humic acid (Cl (PHA)) and peat-derived fulvic acid (Cl (PFA)).

The concentrations of MeHg in the artificial soils are shown in Figure 3, with a value of 4 ng/g in the control. In comparison to the control, there was no significant change in the soil MeHg content of the treatment lacking kaolinite. However, the content significantly increased by 252% in the treatment lacking iron and manganese oxides, suggesting that the presence of iron and manganese oxides could decrease the production of MeHg in the soil. Upon adding FeS_2_ to the control, the MeHg content in the soil significantly increased by 277%. When replacing peat in the soil with fulvic acid and humic acid, the MeHg content decreased, with only the fulvic acid-treated group showing significant differences (*p* < 0.05).

### 3.3. The Concentrations of THg and MeHg in Rice

**Figure 4:**
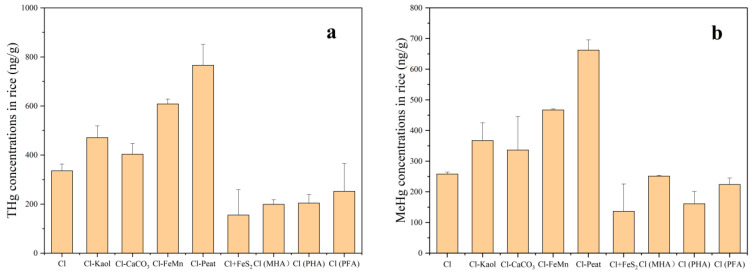
THg concentrations in rice (**a**), MeHg concentrations in rice (**b**). Containing all the components (control group, Cl), removal of kaolinite (Cl-Kaol), calcium carbonate (Cl-CaCO_3_), Fe/Mn oxides (Cl-FeMn) and peat (Cl-Peat); addition of ferrous disulfide (FeS_2_) (Cl+FeS_2_); Replacing peat in the soil with market-derived humic acid (Cl (MHA)), peat-derived humic acid (Cl (PHA)) and peat-derived fulvic acid (Cl (PFA)).

Figure 4 presents the concentrations of THg and MeHg in rice. In the control, the concentrations of THg and MeHg in rice were 336 ng/g and 258 ng/g, respectively. Compared to the control, the soil lacking kaolinite, iron and manganese oxides, and peat had significantly higher the concentration of THg and MeHg in rice (*p* < 0.05). The concentration of THg increased by 40%, 81%, and 128%, respectively, and the concentration of MeHg increased by 42%, 81%, and 157%, respectively. This suggests that the presence of kaolinite, iron and manganese oxides, and peat in the soil can decrease the accumulation of soil THg and MeHg in rice. The absence of CaCO_3_ had no significant effects on both THg and MeHg in rice. The treatment with addition of FeS_2_ had significantly less THg and MeHg in rice (*p* < 0.05), with reduction rates of 54% and 47%, respectively. This indicates that the addition of FeS_2_ can decrease the accumulation of soil Hg in rice. The treatments with fulvic acid and humic acid had less THg in rice, with both humic acid-treated groups displaying significance (*p* < 0.05). The treatment with peat-derived humic acid had less MeHg in rice (*p* < 0.05), while there was no significant difference in the treatments with peat-derived fulvic acid and commercially purchased humic acid compared to the control.

### 3.4. The Concentrations of THg and MeHg in Water Spinach

**Figure 5:**
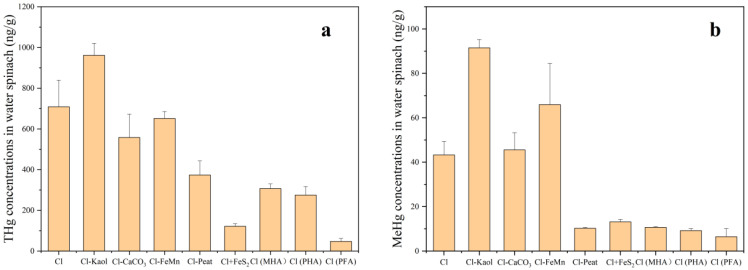
THg concentrations in water spinach (**a**), MeHg concentrations in water spinach (**b**). Containing all the components (control group, Cl), removal of kaolinite (Cl-Kaol), calcium carbonate (Cl-CaCO_3_), Fe/Mn oxides (Cl-FeMn) and peat (Cl-Peat); addition of ferrous disulfide (FeS_2_) (Cl+FeS_2_); replacing peat in the soil with market-derived humic acid (Cl (MHA)), peat-derived humic acid (Cl (PHA)) and peat-derived fulvic acid (Cl (PFA)).

Figure 5 shows the concentrations of THg and MeHg in water spinach. The concentrations of THg and MeHg in the control were 709 ng/g and 43 ng/g, respectively. Compared with the control, the soil lacking kaolinite had significantly higher the concentration of THg (36%) and MeHg (112%) in water spinach. When the soil lacked peat, both THg and MeHg concentrations in water spinach significantly decreased by 47% and 76%, respectively. This suggests that the presence of kaolinite can reduce the accumulation of THg and MeHg in water spinach, while the presence of peat in the soil could enhance the accumulation of THg and MeHg in water spinach. The soil lacking iron and manganese had no significant effect on THg in water spinach but had significantly higher the MeHg concentration (52%) (*p* < 0.05). The absence of CaCO_3_ had no significant effects on both THg and MeHg in water spinach, similar to the case in rice. Following the addition of FeS_2_ to the control, both THg and MeHg concentrations in water spinach were significantly reduced by 83% and 70%, respectively (*p* < 0.05). Upon replacing peat in the soil with fulvic acid and humic acid, both THg and MeHg concentrations decreased significantly (*p* < 0.05). The ranges of reduction were 57% to 93% and 75% to 85%, respectively, with FA treatment having the most significant reduction effect.

## 4. Discussion

### 4.1. The Effects of Soil Texture on Bioavailability of Hg in the Soil

**Figure 6:**
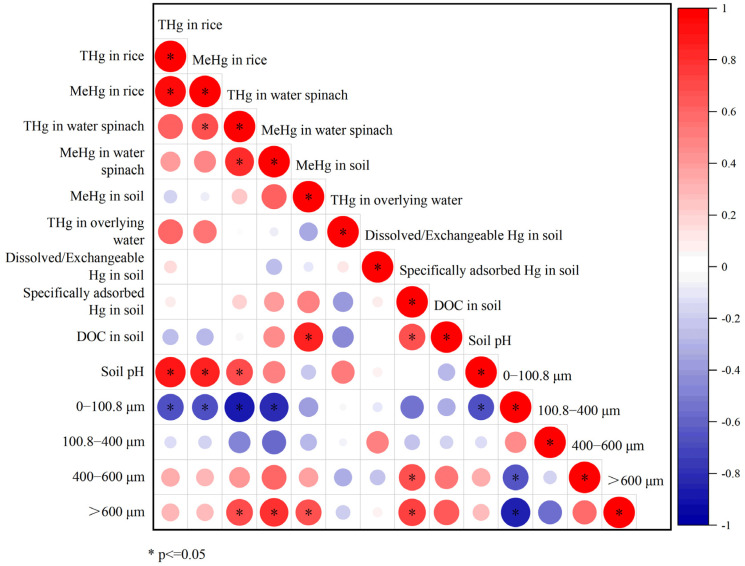
Correlations between plant Hg and soil physicochemical parameters. * represent a significance level at *p* ≤ 0.05.

The results of the study show that different soil components have diverse effects on the bioavailability of soil Hg. Various components primarily influence the physicochemical properties of the soil and the forms of Hg, including soil pH, DOC, soil texture, and Hg species. This subsequently influences the concentration of Hg in plants. The analysis correlating various physicochemical properties with plant Hg concentrations is presented in Figure 6. No significant correlation was observed between the levels of THg and MeHg in the plants and the following parameters: DOC, MeHg, dissolved/exchangeable Hg, and specifically adsorbed Hg in the soils, and THg in the overlying water. However, there was a significant correlation between them and soil particle size. The proportion of particles with a size smaller than 100.8 µm had a significantly negative correlation with THg (*r* = −0.70, *p* < 0.05, *n* = 9), MeHg (*r* = −0.68, *p* < 0.05, *n* = 9) in rice, and THg (*r* = −0.90, *p* < 0.01, *n* = 9), as well as MeHg (*r* = −0.83, *p* < 0.01, *n* = 9) in water spinach. Meanwhile, THg (*r* = 0.72, *p* < 0.05, *n* = 9) and MeHg (*r* = 0.82, *p* < 0.01, *n* = 9) in water spinach had a significantly positive correlation with the proportion of particles with a size larger than 600 µm. Additionally, soil MeHg and specifically adsorbed Hg also had a significantly positive correlation with the proportion of large-sized particles (Figure 6), indicating that, to a certain degree, soil texture may influence the Hg species. It may be also the key factor affecting the absorption and accumulation of THg and MeHg in the plants, especially in water spinach. Some studies have suggested that the bioavailability of metals in soil decreases with a decrease in soil particle size. Heavy metals exhibit higher bioavailability in coarser particles [38,56]. As the particle size of the soil decreases, the specific surface area of the soil increases. Consequently, the soil has a greater capacity to adsorb and complex metals [56,57,58]. Besides soil texture, there was also a correlation between plant Hg and pH, especially in the case of THg (*r* = 0.92, *p* < 0.01, *n* = 9) and MeHg (*r* = 0.87, *p* < 0.01, *n* = 9) in rice. Both had a positive correlation with pH.

Numerous investigations have documented that wetland environments with low pH can activate Hg in the soil and water, thereby promoting the methylation of Hg and its bioavailability [19,59]. However, in this study, while the addition of organic matter reduced the pH, it also reduced the bioavailability of soil Hg due to their adsorption and complexation with Hg. This indirectly establishes a positive correlation between pH and plant Hg. This indicated that in addition to soil texture, the chemical characteristics of soil components themselves can also affect the accumulation of Hg in plants.

### 4.2. The Effects of Soil Inorganic Components on the Methylation and Bioavailability of Hg in the Soil

The results of the pot experiment showed that although the presence of kaolin in the soil could not reduce dissolved/exchangeable Hg, it did lower the levels of MeHg in the soil. It also significantly reduced the concentration of THg and MeHg in rice, as well as in water spinach. Natural clay minerals have a large specific surface area and exhibit strong cation exchange capacity. The surfaces of these minerals contain bonds such as Si-O-Al and C-O. Additionally, they feature active functional groups, including hydroxyl and amidogen. As a result, clay minerals demonstrate a significant adsorption or complexation capacity for metals. However, previous studies have shown that clay minerals like montmorillonite and maifan stone have a limited effect on reducing bioavailable Hg and MeHg in soil [60] and have a slight effect on reducing THg and MeHg in rice and water spinach [60,61]. Compared to that, kaolin can significantly reduce the Hg content in plants, but its mechanism is unclear and further research is needed.

The findings from the pot experiment indicated that the inclusion of iron and manganese oxides in the soil led to a minor increase in the concentration of dissolved/exchangeable Hg. However, there was a significant decrease in the levels of specifically adsorbed Hg and MeHg in the soil. The experiment also demonstrated a reduction in the concentrations of THg and MeHg in rice, as well as MeHg in water spinach. Natural iron and manganese oxides, possessing surface charges and variable-valence elements, can adsorb and complex Hg ions through physical and chemical processes such as surface adsorption, chelation, and redox reactions [62,63], thereby reducing methylation and bioaccumulation of Hg in the soils.

The treatment with addition of FeS_2_ to the soil had significantly higher the concentrations of specifically adsorbed Hg and MeHg in the soil. However, it significantly reduced the concentrations of THg and MeHg in rice and water spinach, indicating a dual effect of promoting the methylation of Hg and reducing Hg accumulation in plants. On the one hand, FeS_2_ can oxidize to form sulfate, thereby enhancing the activity of sulfate-reducing bacteria that can methylate mercury. This leads to an increase in soil MeHg [44]. On the other hand, the addition of FeS_2_ can promote the formation of iron membranes on the surface of plant roots [64], resulting in a reduction in the adsorption of Hg in plants [65]. Furthermore, FeS (s) can not only adsorb mercury ions to form surface compounds of FeS-Hg, but also react with Hg to form β-HgS (s) precipitation, thereby reducing the bioavailability of Hg [66,67]. Meanwhile, the addition of FeS_2_ also raised the proportion of soil with small particles (Figure 1a), thereby enhancing adsorption and fixation of Hg in the soils.

The absence of CaCO_3_ in the soil does not have a significant effect on the concentrations of various forms of Hg in the soil and Hg in plants. This is similar to previous studies, indicating that CaCO_3_ has a weak binding capacity with metals [68], and is not an effective adsorbed component of soil Hg [48], thus weakly affecting the geochemical behavior of Hg.

### 4.3. The Effect of Organic Matter and Its Coupling with Inorganic Components on the Bioavailability of Mercury in Soil

Peat is an organic material that forms in environments characterized by high moisture levels and anaerobic conditions. It primarily comprises partially decomposed plant residues and more decomposed humus. The main components of this humus are fulvic acid, humic acid, and humin. Peat possesses a large surface area and exhibits strong adsorption and chelation capabilities, and is considered both a high-quality adsorbent material and an excellent amendment for soil [69]. Studies have indicated varying effects of peat on Hg-contaminated soil. One study by [70] suggested that adding peat to Hg-contaminated soil could reduce the content of MeHg by 14% and bioavailable Hg by 23%. Contrarily, a study by [45] reported that adding peat to soil can substantially increase the concentration of MeHg. In the present study, the inclusion of peat in the soil showed no significant changes in the concentrations of dissolved/exchangeable Hg, specifically adsorbed Hg, or MeHg in the soil. However, the presence of peat led to a significant reduction in the concentrations of THg and MeHg in rice. Conversely, these concentrations had become higher significantly in water spinach. These observations suggest that different plant species have distinct mechanisms and capacities for absorbing Hg from soil in the same environment. Previous research has demonstrated that metal ions can enter plant root cells through various means, including metal transport proteins, specific or general ion channels, and ion exchange adsorption on the root surface [71,72,73,74]. Additionally, they can combine with low-molecular-weight organic matter in the soil and be utilized as nutrients by plants through absorption [75]. However, studies have shown that the physiological process of metal absorption and enrichment in plants involves many metabolites as well as structural components of cells and tissues, and like the biosynthetic reactions themselves, these processes of transport are under genetic control, owing to disparities in genetics, morphology, anatomy, and other physiological characteristics, distinct plants also display varying capacities to absorb and amass metals [76].

In contrast to peat, humic and fulvic acids led to an overall declining trend in the concentrations of dissolved/exchangeable Hg, specifically adsorbed Hg, and MeHg in the soil. However, most of these changes were not significant. In comparison to the control, the FA-treated group displayed an increase in the concentration of THg in the overlying water, indicating that FA can activate Hg in the soil during the initial stages of cultivation. FA possesses the lowest molecular weight among the components of humic substances (FA, HA, HM), and the highest number of active functional groups, resulting in its greatest complexation capacity for Hg [43,77]. However, the stability of Hg complexation is poor, leading to high environmental activity (volatility in the atmosphere and soil migration) [78,79]. The study by [26] also demonstrated that the addition of exogenous FA to the soil in Hg mining areas significantly enhanced the migration of soil Hg. In comparison with FA, HA is a type of humus with a larger molecular weight, possessing fewer active functional groups and weaker intermolecular repulsion. As a result, HA has a higher capacity to bind with Hg and inhibit the migration of soil Hg, resulting in a lower THg content in overlying water.

Despite FA activating Hg in the soil, compared to the control and the treatment without peat, the treatments with both FA and HA overall reduced the concentrations of THg and MeHg in rice and water spinach. Moreover, the FA-treated group exhibited a significantly greater reduction in THg and MeHg concentrations in water spinach compared to the HA-treated group. On the one hand, FA contains a higher amount of aromatic carbon on its surface compared to HA [77,80], and the greater the aromaticity of DOM, the more challenging it becomes for plants to absorb and utilize [81]. On the other hand, compared to the HA-treated group, there were more soil particles with a size smaller than 100.8 μm in the FA-treated group (*p* < 0.05), which may be more detrimental for bio-utilization of Hg in the soil.

From the preceding discussion, it becomes evident that the interaction of organic and inorganic components can impact the biogeochemical behavior of soil Hg not only through their chemical properties, but also by altering the soil texture. As previously mentioned, soil particle size stands as a crucial factor affecting the bioavailability of soil Hg: smaller soil particles are less conducive to the bioavailability of Hg in the soil. Research has shown that well-developed root systems are often beneficial for plants to uptake soil metals [82], which may result in rice being less influenced by soil texture due to its well-developed root system. In contrast, water spinach with a less developed root system will be more significantly affected by soil texture. Consequently, even though FA activated soil Hg, it significantly enhanced the proportion of soil particles smaller than 100.8 μm, thus inhibiting the accumulation of mercury in plants, particularly resulting in a substantial reduction in the concentrations of THg and MeHg in water spinach. Conversely, the addition of peat to the soil raised the proportion of larger soil particles, leading to a significant rise in Hg levels in water spinach.

## 5. Conclusions

Diverse soil compositions can influence the related physicochemical properties of soil, consequently impacting the methylation and bioavailability of soil mercury. In this study, pot experiments were conducted using artificially created soils with varying components to investigate the effects of different soil constituents, both organic and inorganic, as well as different soil textures resulting from their coupling, on the methylation and bioavailability of Hg. The results reveal that the inorganic components primarily exhibited adsorption and fixation effects on soil Hg. The interaction of organic and inorganic components could impact the biogeochemical behavior of soil Hg not only through their chemical properties but also by altering the soil texture. When contrasted with rice possessing well-developed roots, water spinach with less-developed roots is notably more susceptible to the influence of soil texture. For example, even though FA activated soil Hg, it significantly enhanced the proportion of soil particles smaller than 100.8 μm, thus inhibiting the enrichment of mercury in plants, especially resulting in a substantial reduction in the concentrations of THg and MeHg in water spinach. The presence of peat in the soil, conversely, significantly raised the proportion of soil particles with larger sizes, leading to a noteworthy increase in Hg concentration in water spinach. The results of this study indicate that the effects of different soil components on soil mercury methylation and bioavailability are complex and related not only to the properties of the components themselves, but also to the soil texture formed by the interaction between soil components, as well as related to plant species. When choosing soil amendments for soil mercury pollution control, we need to comprehensively consider these factors. Overall, among inorganic components, iron sulfide has a good effect on reducing plant THg and MeHg, while the stabilizing effect of organic components on soil mercury is closely related to plant species.

## Figures and Tables

**Table 1:** Artificial soil configuration scheme.

Code	Quartzite (kg)	Kaolinite (kg)	Calcium Carbonate (kg)	Iron Oxide (kg)	Manganese Oxide (kg)	Peat (kg)	Humic Acid/Fulvic Acid (kg)	FeS_2_ (kg)
Cl	7.00	4.20	1.40	0.56	1.40	1.50	-	-
Cl-Kaol	11.20	-	1.40	0.56	1.40	1.50	-	-
Cl-CaCO_3_	8.40	4.20	-	0.56	1.40	1.50	-	-
Cl-FeMn	7.70	4.20	1.40	-	-	1.50	-	-
Cl-Peat	7.70	4.20	1.40	0.56	1.40	-	-	-
Cl+FeS_2_	6.92	4.20	1.40	0.56	1.40	1.50	-	0.08
Cl (MHA)	7.00	4.20	1.40	0.56	1.40	-	0.70	-
Cl (PHA)	7.00	4.20	1.40	0.56	1.40	-	0.70	-
Cl (PFA)	4.36	2.40	0.80	0.32	0.08	-	0.04	-

Note: Due to the large differences in the biological availability of each organic component, considering the normal growth of plants, the amount of each organic component is different. Containing all the components (control group, Cl), removal of kaolinite (Cl-Kaol), calcium carbonate (Cl-CaCO_3_), Fe/Mn oxides (Cl-FeMn) and peat (Cl-Peat); addition of ferrous disulfide (FeS_2_) (Cl+FeS_2_); replacing peat in the soil with market-derived humic acid (Cl (MHA)), peat-derived humic acid (Cl (PHA)) and peat-derived fulvic acid (Cl (PFA)).

## Data Availability

Data are contained within the article and supplementary materials.

## Supplementary Materials

The following supporting information can be downloaded at: https://www.mdpi.com/article/10.3390/toxics11110942/s1, Text1: Extraction and purification of humus components; Text2: Chemical analysis [83].
