# Supplementary material for: The Effects of Different Soil Component Couplings on the Methylation and Bioavailability of Mercury in Soil
Source: Toxics. 2023 Nov 20;11(11):942. doi: 10.3390/toxics11110942 (PMC10674802; doi:10.3390/toxics11110942)
Supplement: Supplementary file 1 [file toxics-11-00942-s001.zip › toxics-2677171-supplementary.pdf]

supporting information

The Effects of Different Soil Component Couplings on the Methylation and Bioavailability of Mercury in Soil

Aming Qin [1,2], Shu Ran [1,2], Tianrong He [1,*], Deliang Yin [1], Yiyuan Xu [3]

1. Key Laboratory of Karst Georesources and Environment (Guizhou University), Ministry of Education , Guiyang 550025, China

2. College of Resources and Environmental Engineering, Guizhou University, Guiyang 550025,China

3. State Key Laboratory of Environmental Geochemistry, Institute of Geochemistry, Chinese Academy of Sciences, Guiyang 550081, China

----

* Corresponding author. trhe@gzu.edu.cn

Text1: Extraction and purification of humus components

Extraction

The extraction and purification of HA and FA were performed using a method developed by the International Humic Substances Society (IHSS) [1,2]. Briefly, 100 g of materials (i.e., peat soil) was weighed into a beaker, and added with 1 L 0.1 M NaOH. The solution was intermittently churned for 24 h, and then centrifuged at 15,000 rpm for 20 min to retain supernatant.This process was repeated at least twice to ensure a complete separation of the supernatant from the residue. The pH of supernatant was adjusted to 1 using 6M HCl. HA is insoluble in acidic solutions, while FA is soluble in acid and alkaline solutions. Thus, all of the HA precipitates are separated from the FA. The HA products were obtained by centrifugation for precipitation (5000 rpm, 10 min). All the experiments were carried out at 25℃ conditions.

Purification of FA and HA

The purifications of FA and HA were conducted using a method reported by

[1]. Briefly, the XAD-8 resin (purchased from Sigma Co., USA) was used for the purification of FA. The FA-containing upernatant was collected and flowed through the XAD-8 exchange column, which resulted in the retention of FA on the resin. The resin was then washed with deionized water until pH was 6-7. After that, the FA was eluted from the resin with a 0.1 M NaOH solution. The HA precipitates were dissolved in 0.1M NaOH, then adjusting the pH of solution to 7, and finally dialyzed with deionized water until Cl- was undetectable.

Text2: Chemical analysis

The THg concentrations in the rice grain and water spinach were determined with a DMA-80 analyzer (Milestone, Italy). Briefly, Weigh plant samples 0.01~0.05 g (accurate to 0.0001 g) and place them directly on the injector for the determination of plant THg. The THg concentrations in the overlying water samples were determined by cold vapor atomic fluorescence spectrometry (CVAFS) (Model III, Brooksrand®, USA)using a method described in detail by [3] and [4]. Briefly, For the THg concentrations in the water samples, water samples were oxidized with 0.5% BrCl at least for 24 h. After oxidation, 0.2% $NH_2OH{\cdot}HCl$ was added to destroy the free halogens before adding stannous chloride ($SnCl_2$) to convert divalent Hg to volatile Hg0. The resulting sample was then purged with Hg-free N_2, and Hg(0) was adsorbed onto a gold trap.

The MeHg concentrations in the rice grain, water spinach and soil were determined using methods that have been previously described by [5], [6] and [7]. Approximately 0.3 g of soil sample was placed into a 50-mL centrifuge tube, into which 1.5 mL of 1 M $CuSO_4$, 7.5 mL of 3 M HNO_3, and 10 mL of CH_2Cl_2 were added. The tube was closed and shaken for 30 min. Five milliliters of the CH_2Cl_2 layer was pipetted into another 50-mL centrifuge tube after the tube was centrifuged at 3000 rpm for 30 min. Approximately 40 mL of double-deionized water was added to the tube. The tube was heated at 45 °C in a water bath until no visible solvent was left in the tube. The remaining liquid was then purged with nitrogen for 8 min in a water bath at 80 °C to remove the solvent residue.The sample was brought to 50 mL with double-deionized water before an appropriate volume (generally 15 mL) of the sample was transferred to a borosilicate bubbler. The pH of the collected sample was adjusted to 4.9 using an acetate buffer. The Hg in the sample was ethylated by the addition of sodium tetraethyl borate. The ethyl analog of CH_3Hg,

$CH_3CH_2HgCH_3$, was separated from the solution by purging with N_2 onto a Tenax trap and analyzed by CVAFS. For MeHg in rice grain and water spinach, about 0.15 g of sample was digested using a KOH-methanol/solvent extraction (25%, v / v) technique at a water-bath (80°C) for 3 h. Subsequent extraction, back-extraction and detection were same with the soil MeHg analysis.

The bioavailable Hg contents in the soils were determined with the method of [8], which involved sequential selective extractions of Hg from geological solids. soluble and exchangeable Hg was extracted using 1M $Mg(NO_3)_2$ (adjusted to pH 7 with HNO_3), specifically sorbed Hg was extracted using 1M CHCOONa (adjusted to pH 5 with CHCOOH). The sum of the concentrations of Hg from these two fractions was defined as the bioavailable Hg concentration [9]. The extracted samples were determined using the above method for the analysis of THg in the overlying water。

Soil dissolved organic carbon (DOC) concentration was measured by a TOC analyzer (Vario TOC, Elementar, Germany) at soil: water = 1: 20. The pH was measured by Portable pH meter (SX731, Sanxin, China). Soil particles ware dispersed using water and ultrasonic waves, and their size distributions of the soil were measured by a laser-scattering particle analyzer (Bettersize3000Plus, Bettersize Instruments Ltd., China). The agitator speed was 800r/min and the shading range was 5-15%.

References

1. Zhang, J.; Dai, J.; Wang, R.; Li, F.; Wang, W. Adsorption and desorption of divalent mercury (Hg2+) on humic acids and fulvic acids extracted from typical soils in China. *Colloid Surface A*. **2009**, 335, 194-201.
2. Gondar, D.; Lopez, R.; Fiol, S.; Antelo, J. M.; Arce, F. Characterization and acid–base properties of fulvic and humic acids isolated from two horizons of an ombrotrophic peat bog. *Geoderma*. **2005**, 126, 367-374.
3. Bloom, N. Determination of Picogram Levels of Methylmercury by Aqueous Phase Ethylation, Followed by Cryogenic Gas Chromatography with Cold Vapour Atomic Fluorescence Detection. *Can J Fish Aquat Sci*. **1989**, 46, 1131-1140.
4. He, T.; Feng, X.; Guo, Y.; Qiu, G.; Li, Z.; Liang, L.; Lu, J. The impact of eutrophication on the biogeochemical cycling of mercury species in a reservoir: a case study from Hongfeng Reservoir, Guizhou, China. *Environ Pollut*. **2008**, 154,
5. Liang, L.; Horvat, M.; Cernichiari, E.; Gelein, B.; Balogh, S. Simple solvent extraction technique

for elimination of matrix interferences in the determination of methylmercury in environmental and biological samples by ethylation-gas chromatography-cold vapor atomic fluorescence spectrometry. *Talanta*. **1996**, 43, 1883-1888.

6. Feng, X.; Li, P.; Qiu, G.; Wang, S.; Li, G.; Shang, L.; Meng, B.; Jiang, H.; Bai, W.; Li, Z.; Fu, X. Human exposure to methylmercury through rice intake in mercury mining areas, Guizhou province, China. *Environ Sci Technol*. **2008**, 42, 326-332.
7. Liang, L.; Horvat, M.; Feng, X.; Shang, L.; Li, H.; Pang, P. Re-evaluation of distillation and comparison with HNO3 leaching/solvent extraction for isolation of methylmercury compounds from sediment/soil samples. *Appl Organomet Chem*. **2004**, 18, 264-270.
8. Wang, J.; Feng, X.; Anderson, C. W. N.; Qiu, G.; Ping, L.; Bao, Z. Ammonium thiosulphate enhanced phytoextraction from mercury contaminated soil – Results from a greenhouse study. *J Hazard Mater*. **2011**, 186, 119-127
9. Liu, T.; Wang, J.; Feng, X.; Zhang, H.; Zhu, Z.; Cheng, S. Spectral insight into thiosulfate-induced mercury speciation transformation in a historically polluted soil. *Sci Total Environ*. **2018**, 657, 938-944.
